# Review of *Pseudacrobasis* Roesler, 1975 from China (Lepidoptera, Pyralidae, Phycitinae)

**DOI:** 10.3897/zookeys.615.8859

**Published:** 2016-09-07

**Authors:** Yingdang Ren, Houhun Li

**Affiliations:** 1 Institution of Plant Protection, Henan Academy of Agricultural Sciences, Henan Key Laboratory of Crop Pest Control, Key Laboratory of Integrated Pest Management on Crops in Southern Region of North China, Zhengzhou 450002, P. R. China; 2 College of Life Sciences, Nankai University, Tianjin 300071, P. R. China

**Keywords:** China, Lepidoptera, new species, Pyralidae, Phycitinae, Pseudacrobasis

## Abstract

The genus *Pseudacrobasis* is reviewed for China. *Pseudacrobasis dilatata* **sp. n.** is described as new and compared with *Psorosa tergestella* (Ragonot, 1901). Images of adults and illustrations of genital structures are provided.

## Introduction

*Pseudacrobasis* is a monotypic genus established by Roesler in 1975 for the type species *Pseudacrobasis nankingella* Roesler, 1975 from Nanjing, China. It is widely distributed in China, Korea, Japan, and south of Russian Far East (Roesler 1975; Inoue 1982; Song and He 1997; Bae 2004; Bae, Byun and Paek 2008; Kirpichnikova 1999; Ren and Li 2009, 2012; Yamanaka 2013; Ren 2014), and its discovery in Europe was once thought to be of an alien species (Asselbergs 1998, 2002; Billi 2010; Asselbergs 2002). In 2014, Vives unveiled *Pseudacrobasis nankingella* was a junior synonym of *Psorosa tergestella* (Ragonot, 1901), and transferred *tergestella* (Ragonot, 1901) from *Psorosa* Zeller, 1848 to *Pseudacrobasis* Roesler, 1975, which eliminated “the wrong identification leading to the wrong conclusion on a putatively ‘invasive’ species”. Scalercio (2015) elaborated its biology, ecology, and distribution for the first time.

It has been more than 40 years since the genus establishment, and since then the type species *Pseudacrobasis tergestella* (Ragonot, 1901) is known only. Here, a second species *Pseudacrobasis dilatata* sp. n. is described, based on specimens collected from different localities in China, and it is compared with *Psorosa tergestella* (Ragonot, 1901).

## Material and methods

Genitalia dissections were carried out following the methods introduced by Li (2002). Photographs of adults and venation were taken with a Leica M205A, and photographs of genitalia and details of the head were taken with a Leica DM750, using Leica Application Suite 4.6 software to capture images. The type specimens are deposited in the Insect Collection of Nankai University, Tianjin, China.

## Taxonomy

### Pseudacrobasis

Taxon classificationAnimaliaLepidopteraPyralidae

Roesler, 1975

Pseudacrobasis Roesler, 1975: 100.

#### Type species.

*Pseudacrobasis tergestella* (Ragonot, 1901).

#### Diagnostic characters.

**Figures 1–2.**
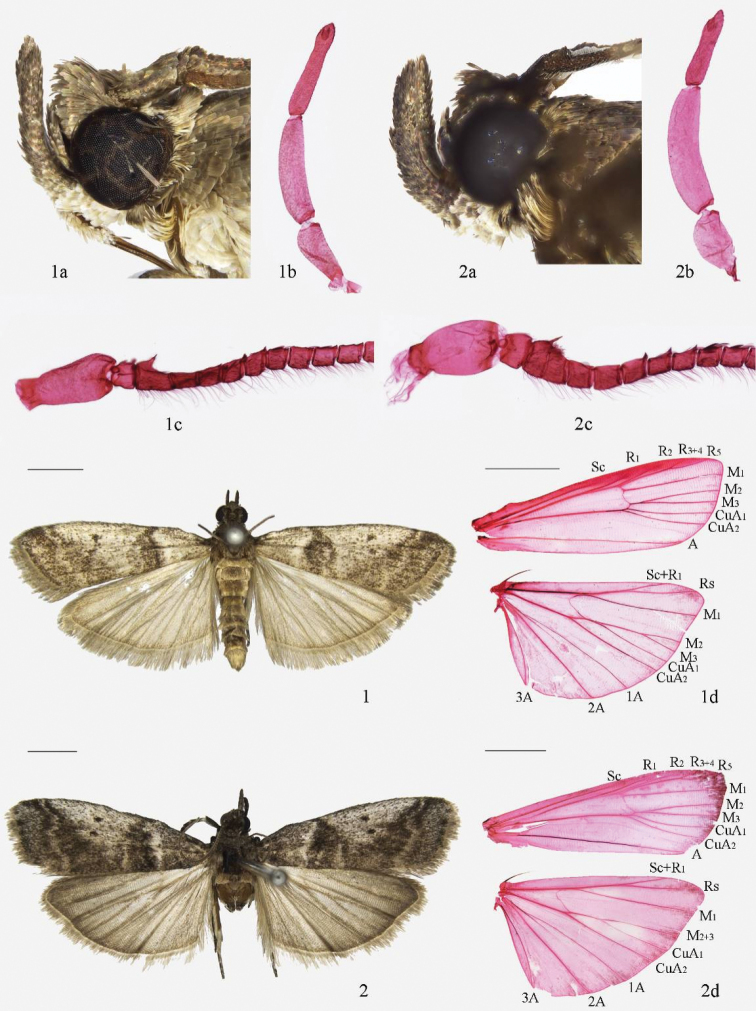
Adult of *Pseudacrobasis* species: **1** *Pseudacrobasis dilatata* sp. n. (**1a** head **1b** labial palpus, slide No. YLL15173 **1c** Antenna, slide No. YLL15173 **1d** wing, slide No. YLL15171w) **2** *Psorosa tergestella* (**2a** head **2b** labial palpus, slide No. YLL15175 **2c** Antenna, slide No. YLL15175 **2d** wing, slide No. LJY10581w). Scale bars: 2.0 mm.

**Figures 3–4.**
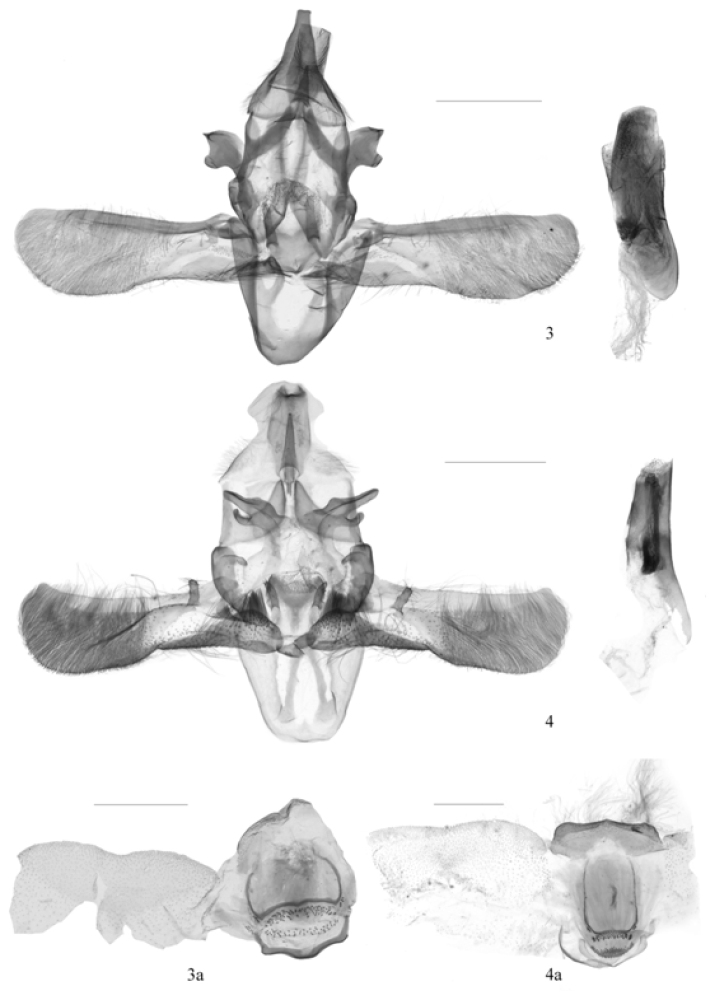
Male genitalia structures of *Pseudacrobasis* species: **3** *Pseudacrobasis dilatata* sp. n. (**3a** culcita), slide No. YLL15176 **4** *Psorosa tergestella* (**4a** culcita), slide No. RYD04497. Scale bars: 0.5 mm.

**Figures 5–6.**
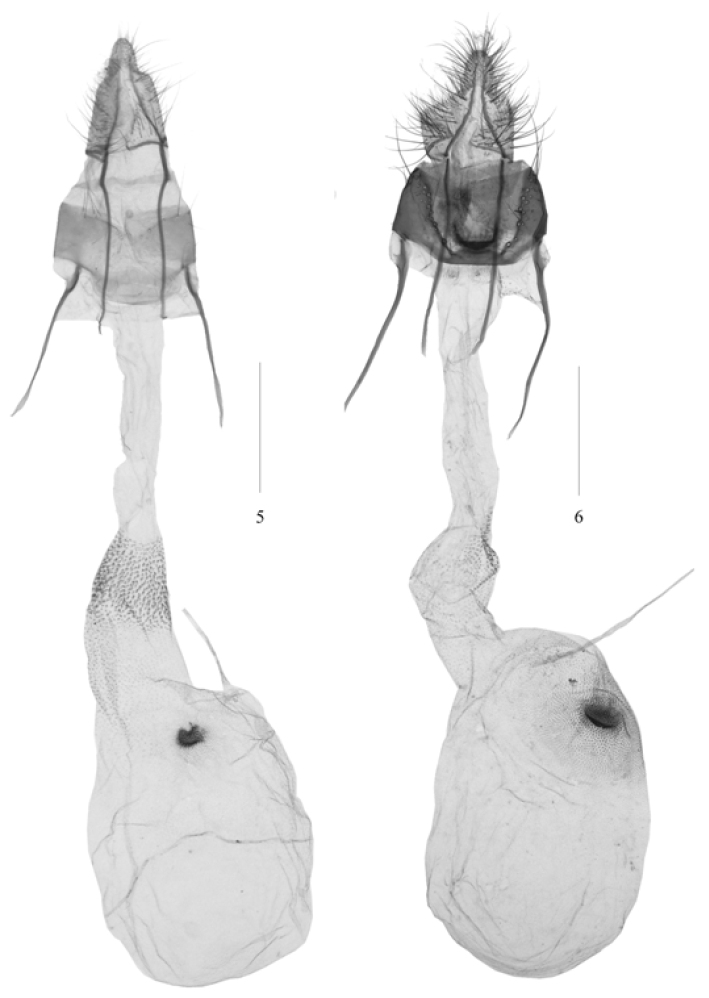
Female genitalia structures of *Pseudacrobasis* species: **5** *Pseudacrobasis dilatata* sp. n., slide No. LHX14109 **6** *Psorosa tergestella*, slide No. YLL15177. Scale bars: 0.5 mm.

*Pseudacrobasis* is characterized by the combination of the following characters: the male antennal scape with a distal scale projection on the inner side, the several basal flagellomeres slightly incurved, forming a shallow sinus containing a smaller scale tuft, the first and several other flagellomeres beyond the sinus bearing a small spine dorsally (Figs 1c, 2c), the ventral surface of the flagellum bearing dense, elongate cilia approximately as long as width of the flagellum, the female antenna simple and weakly pubescent; the labial palpus upturned just beyond the vertex (Figs 1a, 1b, 2a, 2b); the forewing having a fuscous patch on the inner side of the antemedial line and a tuft of scales near the fuscous spot, R_3 + 4_ and R_5_ stalked in basal 2/3, M_2_ and M_3_ very shortly stalked; the hindwing with Rs and Sc stalked for basal 2/5 of Rs, M_2_ and M_3_ long-stalked (Fig. 1d) or fused (Fig. 2d), M_2+3_ shortly stalked with CuA_1_; in the male genitalia, the apical process of the gnathos tapered, the separated transtilla strongly sclerotized, the valva with a small fingerlike clasper at base, the U-shaped juxta with a pair of finger-like lateral lobes, and the phallus with many slightly sclerotized crimples and microtrichia (Figs 3, 4); in the female genitalia, both apophyses anteriores and posteriores of medium length, the former slightly shorter than the latter, the antrum weak-sclerotized or not sclerotized, the membranous ductus bursae with many spinules near the junction with the corpus bursae, the ovate membranous corpus bursae shorter than the ductus bursae, the signum developed as a small, rounded, granulate plate, and the ductus seminalis arising from the corpus bursae posteriorly (Figs 5, 6).

This genus is similar to *Caradjaria* Roesler, 1975 in appearance, but can be distinguished from the latter by the following characters: the male antennal scape with an angular scale process at its inner terminal, which disappears once the scales are removed; the forewing with M_2_ and M_3_ very short-stalked; the apical process of the gnathos tapered, the transtilla separated, and the phallus with sclerotized wrinking and a few minute and weak scobinations in the male genitalia; and the membranous corpus bursae in the female genitalia. In the genus *Caradjaria*, the male antennal scape is enlarged at its inner terminal to form a thorn-like process; M_2_ and M_3_ of the forewing are stalked approximately half of their length; the apical process of the gnathos is enlarged in distal part, the transtilla is connected, and the phallus has small dense spines in the male genitalia; the corpus bursae of the female genitalia is weakly sclerotized in its posterior half.

### Pseudacrobasis tergestella

Taxon classificationAnimaliaLepidopteraPyralidae

(Ragonot, 1901)

Figs 2 , 4 , 6

Psorosa tergestella Ragonot, 1901: 107–108. TL: Italy (Trieste).
Pseudacrobasis nankingella Roesler, 1975: 100. TL: China (Jiangsu).
Pseudacrobasis tergestella (Ragonot, 1901): Vives, 2014: 401.

#### Diagnosis.

Adults (Fig. 2) with wingspan 13.5−18.0 mm. *Pseudacrobasis tergestella* is characterized by the large uncus narrowed from broad base to 3/5, mushroom-like in the distal 2/5; the transtilla distally produced to a stout digitate dorsal process and a curved slender ventral process in the male genitalia (Fig. 4); and by the posterior margin of the eighth tergite deeply concave, U-shaped and with a sclerotized semicircular decoration at middle of anterior 1/3 in the female genitalia (Fig. 6).

#### Distribution.

China (Fujian, Gansu, Guangdong, Guangxi, Guizhou, Hainan, Henan, Hubei, Hunan, Liaoning, Jiangsu, Jiangxi, Jilin, Shaanxi, Shandong, Shanghai, Sichuan, Yunnan, Zhejiang, Taiwan), Korea, Japan, south of Russian Far East, France, Portugal, Italy.

#### Notes.

*Pseudacrobasis tergestella* (Ragonot, 1901) is widely distributed in China. Its identification in this study is based on the examination of 88 male and 67 female specimens. Scalercio (2015) pointed out “the currently known […], the distribution of *Psorosa tergestella* is quite unique with occurrences in the far east and in the far west of the Palearctic region, no records are currently available for Central Asia and East Europe, where suitable habitats are present”. We believe *Psorosa tergestella* is likely to distribute the Central Asian and East Europe region with the depth of the investigation.

### Pseudacrobasis dilatata sp. n.

Taxon classificationAnimaliaLepidopteraPyralidae

http://zoobank.org/AD43828F-F38B-4D24-929D-4F9EC463A172

Figs 1 , 3 , 5

#### Diagnosis.

This new species can be distinguished from its allied species *Psorosa tergestella* by the following characters: M_2_ and M_3_ of the hindwing stalked for approximately 3/5 of their lengths; in the male genitalia by the subtriangular uncus gradually narrowed to truncate apex, the transtilla with its distal part developed into two small horns of nearly equal sizes; in the female genitalia by the posterior margin of the eighth tergite only slightly concave and lacking decoration. In *Psorosa tergestella* (Figs 2, 4, 6), M_2_ and M_3_ of the hindwing are completely fused (Fig. 2d); in the male genitalia, the uncus is mushroom-like in distal 2/5, the distal part of the transtilla is developed as one large and one small horns (Fig. 4); in the female genitalia, the posterior margin of the eighth tergite is deeply concave and U-shaped, and has a sclerotized semicircular mark in the middle of the anterior 1/3 (Fig. 6).

#### Description.

Adult (Fig. 1): Wingspan 14.0−19.0 mm. Head (Fig. 1a) greyish brown. Antenna (Fig. 1c) with scape greyish brown dorsally, greyish white ventrally, flagellum greyish brown. Labial palpus (Fig. 1b) with first segment greyish white, second and third segments about equal length, greyish brown. Patagium, tegula and thorax greyish brown. Forewing greyish brown, densely dusted with white; basal 1/4 greyish white; antemedial line brown, curving obliquely from basal 1/4 of costa to 2/5 of termen, bordered inwardly by a rounded-triangular fuscous patch, edged with a black ridge of raised scales on inner side, edged inwardly with white; large, triangular, greyish white blotch between antemedial and postmedial lines, reaching near dorsum; discal spots brownish fuscous, rounded, distinct and separated; postmedial line grayish white, sinuate, concave at M_1_ and CuA_2_, edged with brown; terminal line black; cilia greyish white basally, light brown distally. Hindwing greyish white, with ten veins, M_2_ and M_3_ stalked for about 3/5 of their length; termen and cilia light gray. Legs brownish fuscous dusted with greyish white, tarsomeres white at end. Abdomen greyish brown, each segment yellowish brown at end.

Male genitalia (Fig. 3). Uncus subtriangular, broad in basal half, distinctly narrowed at middle, then gradually narrowed to truncate apex. Gnathos with apical process triangular, about 2/5 length of uncus, shorter than lateral arm; lateral arm widely banded, slightly narrowed distally. Transtilla with basal 2/3 narrowed, clubbed, distal 1/3 dilated, stout; apex concave medially, forming a small horn-shaped dorso- and ventro-apical process of about equal size. Valva three times as long as wide, distal 3/5 densely setose, broader than basal 2/5; clasper placed near costa at basal 1/5 of valva, small, fingerlike; costa a narrowed club, extending to end of valva; sacculus fusiform, about 1/3 length of valva, bearing long stouter setae. Juxta nearly quadrate, slightly concave at middle of posterior margin; lateral lobe finger-like, as long as apical process of gnathos, with sparse setae distally. Vinculum U-shaped, moderately long with transverse posterior margin. Phallus cylindrical, about same length as valva, with sclerotized crimples, granulate distally.

Female genitalia (Fig. 5). Anal papillae subtriangular, narrowed in distal 1/4, rounded at apex. Eighth abdominal segment collar-shaped, approximately 2.5 times wider than long, with tergite arched at middle of anterior margin, slightly concave on posterior margin. Apophyses posteriores slightly longer than apophyses anteriores. Antrum oval, weak-sclerotized. Ductus bursae with posterior 3/5 smooth, moderate in width, anterior 2/5 gradually broadened, with dense spinules from anterior 1/5 to 2/5. Corpus bursae ovate, about 3/4 length of ductus bursae, punctate near signum; signum at posterior 1/5, consisting of concentrically arranged minute scobination, shallowly concaved in middle. Ductus seminalis attached to posterior margin of corpus bursae.

#### Material examined.

Holotype ♂, China: Shaanxi, Danfeng, Tieyupu, (33.63°N, 110.53°E; elevation 680 m), 28 May 1994, leg. Jin Zhou. Paratypes: 1 ♀, Gansu, Wenxian, Bifenggou, (32.95°N 104.67°E; elevation 860 m), 10 July 2005, leg. Hai-Li Yu; 1 ♂, Guizhou, Chishui, Suoluo, (28.44°N, 106.03°E; elevation 390 m), 27 May 2000, leg. Yan-Li Du; 4 ♀♀, Guizhou, Xishui, Linjiang, (28.21°N, 106.18°E; elevation 500 m), 3 June 2000, leg. Yan-Li Du; 1 ♂, Guizhou, Fanjingshan, Heiwan, (27.94°N, 108.61°E; elevation 530 m), 2 June 2002, leg. Xin-Pu Wang; 3 ♂♂, Guizhou, Daozhen, Dashahe, (28.87°N, 107.61°E; elevation 600 m), Xiannvdong, 28 May 2004, leg. Shu-Lian Hao; 1 ♂, Guizhou, Daozhen, Dashahe, (28.87°N, 107.61°E; elevation 600 m), Xiannvdong, 17 August 2004, leg. Yun-Li Xiao; 2 ♂♂, Guizhou, Daozhen, (28.87°N, 107.61°E; elevation 1300 m), Chengjiashan, 19 August 2004, leg. Yun-Li Xiao; 1 ♂, Hebei, Jingxing, Mt. Xiantai, (38.12°N, 113.84°E; elevation 100 m), 23 July 2000, leg. Hai-Li Yu; 4 ♂♂, Henan, Huixian, Baligou, (35.59°N, 114.00°E; elevation 780 m), 12 July 2002, leg. Xin-Pu Wang; 3 ♂♂, Henan, Huixian, Guanshan, (35.50°N, 113.59°E; elevation 550 m), 25−26 July 2006, leg. Deng-Hui Kuang, Hui Zhen; 2 ♂♂, Henan, Jiyuan, Wangwushan, (35.15°N, 112.28°E; elevation 1100 m), 30 July 2006, leg. Deng-Hui Kuang, Hui Zhen; 2 ♂♂, Henan, Yiyang, Huaguoshan, (34.34°N, 111.89°E; elevation 1000 m), 1 August 2006, leg. Deng-Hui Kuang, Hui Zhen; 4 ♂♂, 1 ♀, Hubei, Shennongjia, Bajiaomiao, (31.76°N, 110.57°E; elevation 1100 m), 19 July 2003, leg. Shu-Lian Hao; 1 ♀, Hubei, Shennongjia, (31.34°N, 110.57°E; elevation 1700 m), Wenquan, 21 July 2003, leg. Shu-Lian Hao; 1 ♂, Hubei, Shennongjia, Songbaizhen, (31.75°N, 110.66°E; elevation 1200−1400 m), 17 July 2003, leg. Shu-Lian Hao; 1 ♂, Qinghai, Xunhua, Mengda, (35.83°N, 102.69°E; elevation 2240 m), 15 July 1995, leg. Hou-Hun Li, Shu-Xia Wang. 2 ♂♂, 3 ♀♀, Shaanxi, Yangling, (34.27°N, 108.08°E; elevation 450 m), 3−11 June 1985, leg. Hou-Hun Li; 4 ♂♂, 6 ♀♀, same data as holotype; 1 ♀, Shaanxi, Baihe, Qianpo, (32.81°N, 110.11°E; elevation 200 m), 16 May 1994, leg. Jin Zhou; 51 ♂♂, 47 ♀♀, Shanxi, Jincheng, Lingchuan, Xizhashuicun, (35.78°N, 113.28°E; elevation 900 m), 12−18 July 2010, leg. Hai-Yan Bai, Lin-Lin Yang; 1 ♂, Sichuan, Jianyang, Pingquan, (30.34°N, 104.64°E; elevation 350 m), 4 May 1994, leg. Jin Zhou; 1 ♀, Sichuan, Mabian, Yonghong, (28.55°N, 103.42°E; elevation 1200 m), 22 July 2004, leg. Ying-Dang Ren; 2 ♂♂, Sichuan, Tianquan, Lamahe, (30.35°N, 102.42°E; elevation 1300 m), 29 July 2004, leg. Ying-Dang Ren; 2 ♂♂, Zhejiang, Mt. Jiulong, (28.21°N, 118.68°E; elevation 400 m), 4−5 August 2011, leg. Lin-Lin Yang, Na Chen.

#### Distribution.

China (Gansu, Guizhou, Hebei, Henan, Hubei, Qinghai, Shaanxi, Sichuan and Zhejiang).

#### Etymology.

The specific name is derived from the Latin *dilatatus* (dilate), referring to the dilated distal part of the transtilla.

## Supplementary Material

XML Treatment for Pseudacrobasis

XML Treatment for Pseudacrobasis tergestella

XML Treatment for Pseudacrobasis dilatata

## References

[B1] Asselbergs JEF (1998) *Pseudacrobasis nankingella* Roesler, 1957. An east-asiatic species found in Spain (Lepidoptera: Pyralidae, Phycitinae). SHILAP Revista de Lepidopterología 26(101): 41–43.

[B2] Asselbergs JEF (2002) Données sur les captures recentes dans le sud-ouest de l’Europe de *Pseudacrobasis nankingella* Roesler, 1975, Phycite originaire de l’Extreme-Orient (Lepidoptera, Pyralidae, Phycitinae). Alexanor 21: 491–494.

[B3] Bae YS (2004) Superfamily Pyraloidea II (Phycitinae & Crambinae etc.). Economic Insects of Korea 22. Insecta Koreana Suppl. 29. Junghaeng-Sa, Seoul, 207 pp.

[B4] Bae YS Byun BK Paek MK (2008) Pyralid Moths of Korea (Lepidoptera: Pyraloidea). Korea National Arboretum. Samsungad Com, Seoul, 426 pp.

[B5] Billi F (2010) *Pseudacrobasis nankingella* Roesler, 1975 in Alpes-Maritimes (Lepidopteres Pyralidae). Riviera Scientifique 94: 89−90.

[B6] Corley MFV Merckx T Cardoso JP Dale MJ Marabuto E Maravalhas E Pires P (2012) New and interesting Portuguese Lepidoptera records from 2011 (Insecta: Lepidoptera). SHILAP Revista de Lepidopterología 40(160): 489–511.

[B7] Inoue H (1982) Pyralidae. In: Inoue H Sugi S Kuroko H Moriuti S Kawabe A Owada M (Eds) Moths of Japan. Kodansha, Tokyo 1: 307−404; 2: 233−254.

[B8] Li HH (2002) The Gelechiidae of China (I) (Lepidoptera: Gelechioidea). Nankai University Press, Tianjin, 504 pp.

[B9] Ragonot EL Hampson GF (1901) Monographie des Phycitinae et des Galleriinae. In: Romanoff NM (Ed.) Mémoires sur les Lépidoptères VIII 8 St. Petersburg, 602 pp.

[B10] Ren YD Li HH (2009) Phycitinae. In: Li HH et al. (Eds) Insect Fauna of Henan (Lepidoptera: Pyraloidea). Science Press, Beijing, 57−142.

[B11] Ren YD Li HH (2012) Phycitinae. In: Li HH et al. (Eds) Microlepidoptera of Qinling Mountains (Insecta: Lepidoptera). Science Press, Beijing, 288−417.

[B12] Ren YD (2014) Order Lepidoptera. In: Shen XC et al. (Eds) Insect fauna of Henan catalogue and distribution. Science Press, Beijing, 605−933.

[B13] Roesler RU (1975) Phycitinen- Studien XI, Neue Phycitinae aus China und Japan (Lepidoptera: Phycitinae). Deutsche Entomologische Zeitschrift (N. F.) 22: 79−112.

[B14] Scalercio S Slamka F (2015) Wrong taxonomy leads to a wrong conclusion on a putatively ‘invasive’ species to Europe: the case of *Pseudacrobasis nankingella* (Lepidoptera Pyralidae). Redia XCVIII, 13–19.

[B15] Song SM He MS (1997) Pyralidae. In: Yang XK (Ed.) Insects of the Three Gorge Reservoir Area of Yangtze River. Chongqing Press, Chongqing, 1096−1220.

[B16] Yamanaka H (2013) Phycitini. In: Hirowatari T Nasu Y Sakamaki Y Kishida Y (Eds) The Standard of Moths in Japan. Gakken Education Publishing, Tokyo 4: 335−368.

[B17] Kirpichnikova BA (1999) Lepidoptera: Pyraloidea. In: Lelej AS et al. (Eds) Key to the insects of Russian Far East, Vol. V. Trichoptera and Lepidoptera, Part 2. Dal’nauka, Vladivostok, 443–496.

[B18] Vives MA (2014) Systematic and synonimic catalogue of Lepidoptera of the Iberian Peninsula, of Ceuta, of Melilla and of the Azores, Balearic, Canary, Madeira and Savages Islands (Insecta: Lepidoptera). SHILAP Revista de Lepidopterología, (Supplement), 1184 pp.

